# PVD for Decorative Applications: A Review

**DOI:** 10.3390/ma16144919

**Published:** 2023-07-10

**Authors:** Mariya Vorobyova, Fabio Biffoli, Walter Giurlani, Stefano Mauro Martinuzzi, Maximilian Linser, Andrea Caneschi, Massimo Innocenti

**Affiliations:** 1 Department of Chemistry “Ugo Schiff”, University of Florence, Via della Lastruccia 3, 50019 Sesto Fiorentino, Italy; 2 National Interuniversity Consortium of Materials Science and Technology (INSTM), Via G. Giusti 9, 50121 Firenze, Italy; 3 L.E.M Industries S.p.A, Via Leo Valiani 45/47, 52021 Bucine, Italy; 4 Department of Industrial Engineering (DIEF), University of Florence, Via Santa Marta 3, 50139 Firenze, Italy; 5 CNR-ICOMM, Insititute of Chemistry of Organometallic Compounds, National Research Council (CNR), Via Madonna del Piano 10, 50019 Sesto Fiorentino, Italy; 6 CSGI (Center for Colloid and Surface Science), Via della Lastruccia 3, 50019 Sesto Fiorentino, Italy

**Keywords:** PVD, coatings, fashion, decorative, thin-film characterization, sputtering, magnetron, physical vapor deposition

## Abstract

Physical Vapor Deposition (PVD) is a widely utilized process in various industrial applications, serving as a protective and hard coating. However, its presence in fields like fashion has only recently emerged, as electroplating processes had previously dominated this reality. The future looks toward the replacement of the most hazardous and toxic electrochemical processes, especially those involving Cr(VI) and cyanide galvanic baths, which have been restricted by the European Union. Unfortunately, a complete substitution with PVD coatings is not feasible. Currently, the combination of both techniques is employed to achieve new aesthetic features, including a broader color range and diverse textures, rendering de facto PVD of primary interest for the decorative field and the fashion industry. This review aims to outline the guidelines for decorative industries regarding PVD processes and emphasize the recent advancements, quality control procedures, and limitations.

## 1. Introduction

**Figure 1:**
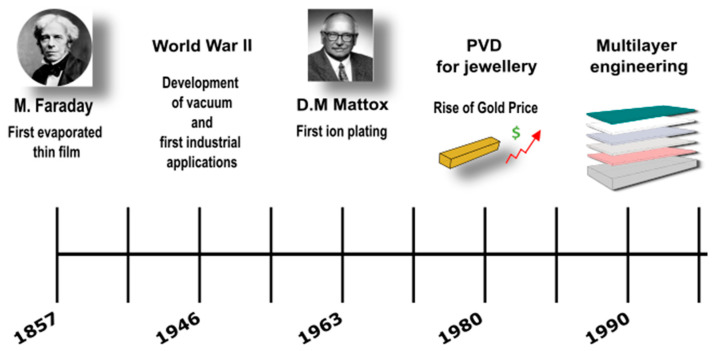
Historical timeline of thin-film evolution connected to the most relevant events, which highlights the significant innovations in PVD techniques.

The physical vapor deposition (PVD) process has been applied since the early 1900s, but most of its development occurred in the 1960s and 1970s, becoming extensively utilized in industrial applications (Figure 1). Advancements in vacuum technology and deposition techniques have expanded the range of substrates to which PVD thin films can be applied, allowing for greater control over the properties of these films. Today, PVD technology is still to be improved; new materials, new techniques, as well as cost reduction have enabled applications in sectors such as the aerospace [1], automotive [2], electronics [3] and even fashion industry [4]. Specifically, the fashion industry has recently embraced PVD to develop more sustainable manufacturing processes. This review aims to overview both the state-of-the-art of PVD and the research perspectives.

The first evaporated thin films can be traced back to Faraday’s experiment in 1857 [5], where he evaporated metal wires in a vacuum. Subsequently, in 1887, Nahrwold [6] replicated the deposition of thin metal films in a vacuum, following Joule’s heating experiments. A year later, Kundt [7,8] measured the refractive indices of such films. The application of this technology on an industrial scale had to wait for the development of vacuum techniques, which emerged after World War II, around 1946. The exponential growth rate of thin films is well-documented in Olang’s excellent review of deposited films in the *Handbook of Thin Film Technology*, as well as Holland’s classic textbook [8] reflecting the substantial pioneering work performed by the author.

The ion plating technique was first described by Mattox [9] in 1963, although a similar technique had previously been reported by Berghaus, who claimed that the coating had “a perfect structure and adhering strength”, even for thicker layers. It was Mattox that stimulated considerable interest and spurred the development of these techniques. Mattox asserted that the technique produced films with exceptional adhesion, even when the film and substrate materials were mutually insoluble. Moreover, the films exhibited thick uniformity and effectively coated irregular surfaces. Many subsequent investigations have since confirmed these findings.

The first metal deposits realized through glow discharge plasma were reported by Grove in 1852 [8]. In 1980, sputtering and its applications experienced rapid growth, with advancement in the apparatus, process modifications, scientific understanding, and expanded application areas, similar to other PVD techniques.

In 1961, two independent US universities initiated research on high deposition rates and full-dense PVD coatings on self-supported shapes. At the Lawrence Livermore Laboratory of the University of California, Bunshah and Juntz [10] produced high-purity beryllium foil and titanium sheet and characterized them in terms of impurity content, microstructure, and mechanical properties. During the same years, Smith and Hunt at Temescal Metallurgical Corporation in Berkeley, California focused on the depositing of various metals, alloys, and compounds.

In the early 1960s, USSR scientists began their work on thin- and thick-film deposition at the Kharkiv Polytechnique Institute, and later at the Paton Electric Welding Institute in Kiyv. Between 1962 and 1969, various steel companies dedicated significant efforts to producing Al and Zn coatings on steel using PVD processes on a production scale [11].

In 1969, Airco Temescal Corporation [12] successfully manufactured Ti-6AZ-4V alloy foil. Pilot production quantities were adopted in honeycomb structures on the SST aircraft, although the aircraft project ultimately failed. The production capability was impressive, with a production rate of 1200 ft per run of Ti-6AQ-4V foil, 12″ wide and 0.002″ thick at a rate of 2–3 ft per minute; moreover, the cost was significantly lower than rolled material.

Studies of thin films had advanced before thick- and bulk-film technology, as documented by Bunshah [13] and Paton, Movchan, and Demchishin [14]. Soviet literature also includes numerous references to the great work on thin and thick films by Palatnik of the Kharkiv Polytechnique Institute. The development of ion plating and sputtering processes continued progressing rapidly and interactively, repeating substantial benefits from their shared characteristics [15].

Towards the end of the 1970s [16], the escalating cost of gold prompted the jewelry industry to explore alternatives in realizing micrometer-thick gold coatings. This led to the replication of golden coatings using TiN PVD, which proved to be a cost-effective solution for decorative purposes. While PVD processes had traditionally been employed for wear-resistant coatings [17] on cutting and forming tools, they also demonstrated a potential solution for decorative coatings. PVD processes offer several advantages compared with electrochemical methods. One advantage is the ability to produce abrasion-resistant coatings with a variety of shades of golden color. But the most appealing advantage of PVD processes is their greater environmental friendliness and sustainability [18,19]. Figure 1 illustrates a historical timeline highlighting significant events in developing PVD coatings. Towards the late 1990s, Préci-Coat [20] achieved the first reported industrialization of yellow PVD coating for high-end vogue applications.

Since the 2000s, research efforts have focused on improving coatings’ characteristics, such as adhesion, corrosion, wear resistance, and deposition rate, as discussed extensively in detail by Baptista et al. in 2018 [21]. In the last five years, companies have focused on reducing production costs, making processes more energy-efficient, lowering material consumption, and recovering precious metals [22]. The most recent advancements in multilayer engineering coatings were clearly described in the brief review by Liu et al. in 2022 [23].

The fashion industry is characterized by a dynamic and ever-changing nature. Short production lead times, low production volumes, high price volatility, low workflow predictability, impulsive purchasing, and wide variability in aesthetic requirements are common issues faced by companies operating in this sector. The manufacturers must frequently adjust their offering by altering the colors of raw materials and components, making the finishing process a critical aspect of production. Fashion products must reflect the current mood and trends, resulting in a brief and seasonal selling period that may last only a few months or weeks. External factors such as weather conditions and customer preferences can influence demand for fashion products, leading to unpredictable sale volumes.

Metal accessories, including closures, buckles, rings, loops, and clasps, play a crucial role in designing and realizing a fashion product. These accessories are typically made from copper alloys (e.g., brass) or steel, and then they are coated with a thin layer of precious metal such as gold, ruthenium, or palladium. The traditional finishing process for metallic items in the fashion industry involves electroplating, which is widely used in countries like Italy and France, where the most important companies are based. Although recently introduced in this production sector, PVD technology has played a secondary role [4].

**Figure 2:**
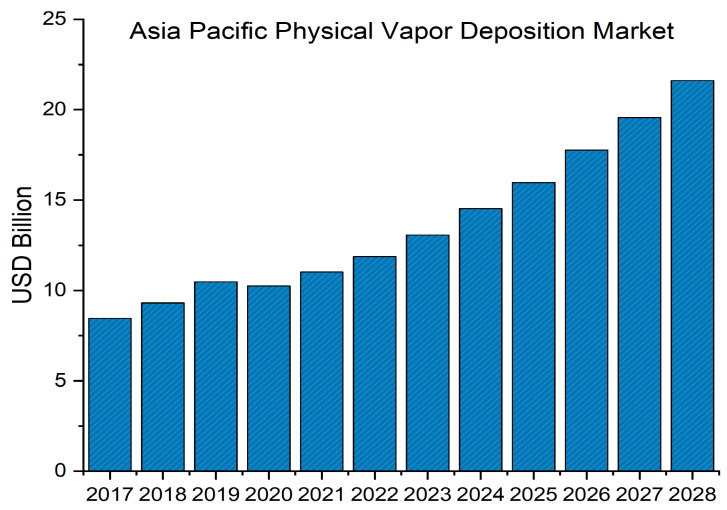
*Fortune Business Insights* estimation for the PVD market in the Asia Pacific Market for 2028, data from [24].

*Fortune Business Insights* [24] valued the global physical vapor deposition market size at USD 22.43 billion in 2020, with projections indicating reaching USD 40.97 billion at a compound annual growth rate (CAGR) of 8.2% from 2021 to 2028. Figure 2 provides an estimation of the market size in the Asia Pacific region, demonstrating the rising demand for PVD products.

**Figure 3:**
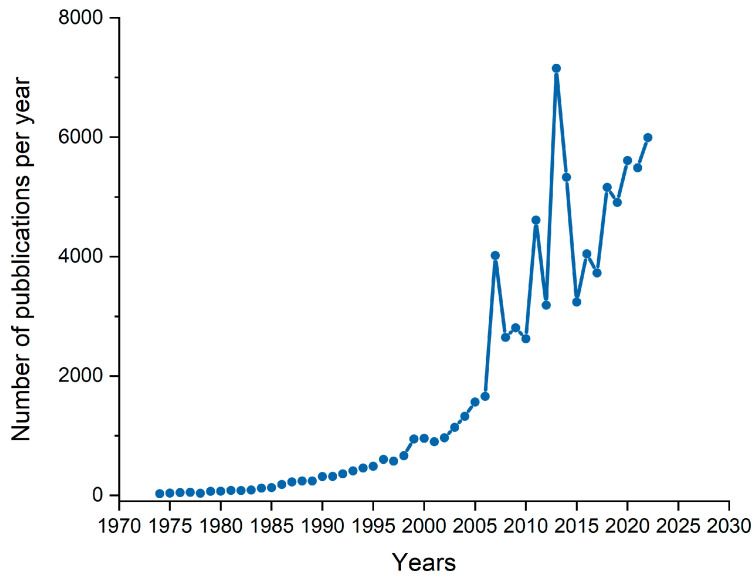
Number of publications per year from 1974 to 2023 on the “PVD” topic, data from [37].

Traditionally, electroplating has been utilized to obtain protective coatings in the jewelry industry. However, this approach carries significant environmental consequences, including generating toxic heavy metals, gases, and harmful waste [25,26]. Recently, there has been a growing focus on investigating electroplating technology [27,28,29,30,31] due to its high environmental impact. Electroplating effluents contain highly toxic substances such as cyanides and metal ions, making wastewater treatment technologically complex and expensive. Consequently, businesses in the fashion and design industry, as well as the faucets [32] and tiles industry [33,34], are seeking and investing in alternative solutions. Several advantages, including a lower environmental impact and removing the need for wastewater treatments, make PVD an appealing alternative to electrodeposition [35]. A comparative study conducted by Martinuzzi in 2022 highlighted the advantages and disadvantages of both techniques [36]. As is shown in Figure 3, there is a clear and continuous increase in the general interest and the adoption of PVD coatings [37].

This review aims to provide a comprehensive summary of the latest scientific publications and recent developments in the field of PVD coatings, specifically focused on their industrial decorative applications. The primary objective is to present a thorough comparative analysis of traditional galvanic coating processes and the more eco-friendly alternatives offered by PVD technologies. The review highlights the shortcomings of conventional methods and proposes potential replacements, suggesting specific industrial production processes. Furthermore, the review describes industrial coating processes presenting the most common substrate materials (e.g., brass and steel). It then details the substrate preparation procedures and coatings characterization, including tests for color and brightness. Finally, this review covers all aspects of quality control processes for decorative purposes. The authors aim to present the untapped potential of PVD technology and try to dispel any stereotypes and mistrust surrounding its use.

## 2. Fundamentals of Physical Vapor Deposition (PVD)

Physical Vapor Deposition is a widely used technique for depositing thin films, both in basic and applied research, as well as in various industrial sectors. It finds extensive uses in realizing protective coatings for biomedical applications and in developing thermal barrier systems (especially for aircraft engines) [38,39], in optics [40], and in electronic components manufacturing [41]. These coatings can improve the substrate’s performance by increasing hardness, wear [42], and corrosion resistance; improvements in tribological [43], optical, and electrical properties are also reported. A combination of these properties is often required [35].

PVD is a vacuum-based technique that involves depositing a thin layer or multilayers of target materials onto a substrate. The thickness of coatings ranges from a few angstroms to several microns, and the deposition rate depends on various operating parameters, including the total pressure in the chamber [44], the partial pressures of the carrier and the reactive gases [45], power and source of usage [46], voltage biases [47], temperature [48], and the sputtering yield of the target [49]. It is important to note that in industrial practice depositing hard coatings is just one step in a sequence of operations that includes the mechanical preparation of surfaces, cleaning, heating, etching, coating, cooling, and conditioning; the weakest link in this chain, therefore, defines the overall quality of the coating. In some cases, specific steps may be skipped; however, this does not make things easier because methods and operating parameters for the remaining steps vary significantly depending on the coating material, the process used, and the desired final performance specifications [19].

**Figure 4:**
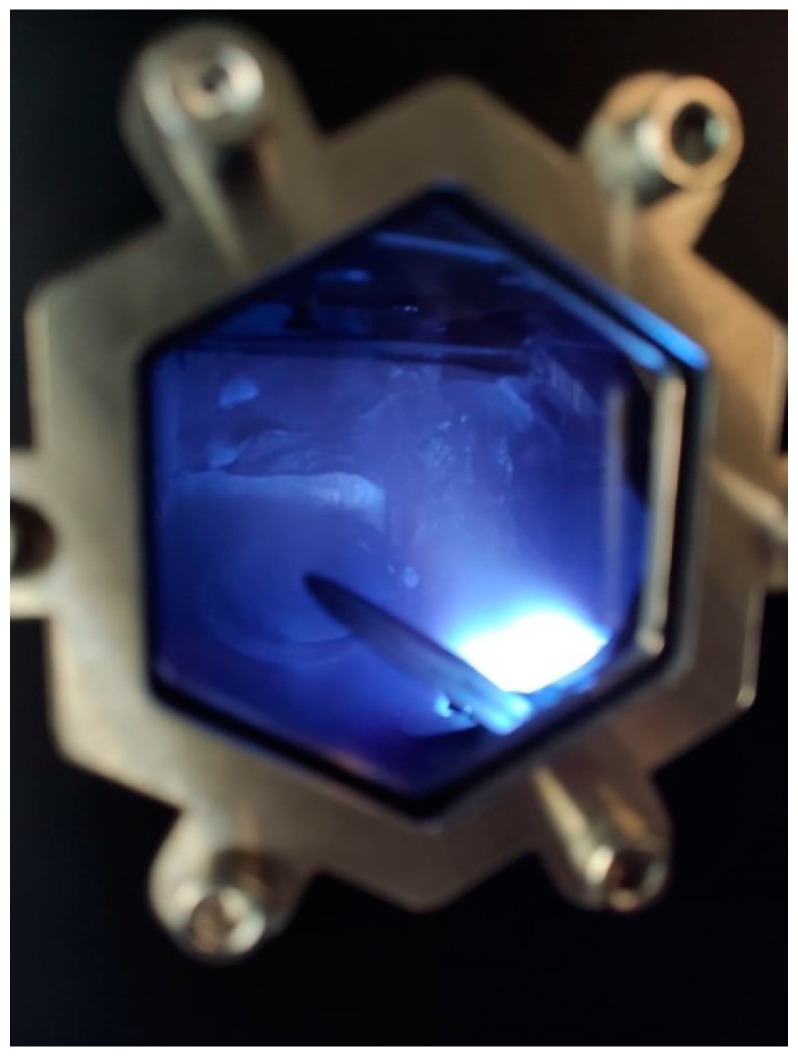
Example of plasma production in magnetron sputtering system.

During the deposition process, the material is vaporized from a solid into an atomic or molecular form and transported through a vacuum or low-pressure gas environment or plasma (Figure 4) towards the substrate, where it condenses. Vacuum deposition typically takes place in a chamber with a pressure range of 10^−5^ Torr to 10^−9^ Torr, depending on the level of gas contamination that can be tolerated. The rate at which deposition occurs ranges from 10 Å/s to 100 Å/s and it can be influenced by many factors, such as evaporation or the sputtering yields of materials. For instance, gold has a higher deposition rate than chromium under the same conditions.

Additionally, the deposition rate is lowered by improving the gas pressure via the addition of both carrier gas (Ar is the most used for this purpose) and reactive gas (O_2_, N_2_, acetylene), increasing their flow rate (expressed in Sccm, Standard cubic centimeters per minute), or through target poisoning, which can create a hysteresis effect [50,51]. A proper adjustment in the voltage bias and (sample heating) temperature can produce better film properties, such as high density and improved adhesion [52,53].

Over the years, the advancement of technologies in the construction of automated production plants has allowed the scale-up of various PVD deposition techniques [54]. In the following sections, the most widely used in industry will be presented and subdivided according to the main physical phenomenon on which they are based: evaporation or sputtering.

### 2.1. Evaporation Systems

**Figure 5:**
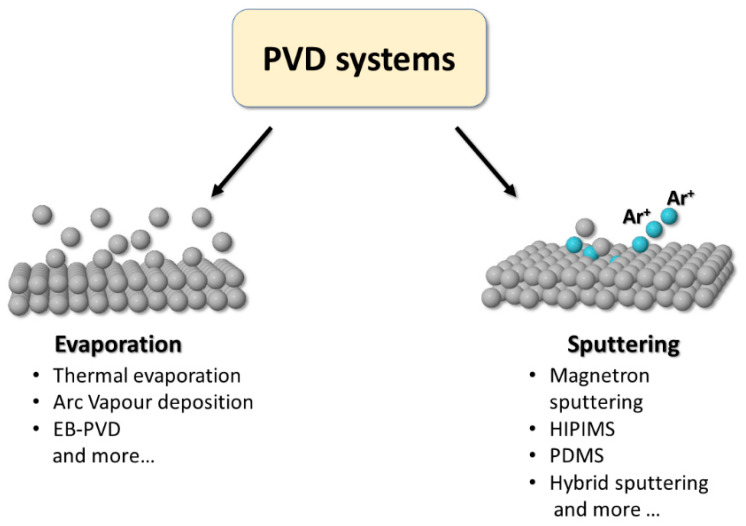
Schematic representation of most relevant physical vapor deposition techniques grouped by process type.

Evaporation systems are based on an apparatus in which the material is transferred from a heat source to the substrate with little or no interaction with the gas molecules in the chamber. A schematic representation of the most common techniques are reported in Figure 5 on the left side; all share the “line-of-sight” trajectory for the evaporated particles [55]. In evaporating alloys, according to Raoult’s law, the vapor’s composition (and, therefore, that of the coating) reflects the relative vapor pressures of the components. Arc Vapor Deposition [56] involves the use of a high-current, low-voltage arc to vaporize a cathodic electrode (cathodic arc) or anodic electrode (anodic arc) and deposit the vaporized material onto a substrate. A voltage bias is often applied to the substrate’s holder, accelerating the ionized source material in its direction. This method is commonly used to deposit both decorative and hard coatings. The need to reduce the structural defects of the coatings drove the development of combined systems like Arc Ion Plating [57], also known as Ion-Assisted Deposition (IAD) or Ion Vapor Deposition (IVD). In ion plating processes, the substrate is bombarded with highly energetic ions before and during the deposition; this can increase the density of the deposited coating, limiting defects during growth. The ions are generated throughout the deposition by heat or by a plasma source. Another popular evaporation method is Electron Beam PVD (EB-PVD); here, electrons from an electron gun are focalized by magnetic fields on the coating material, producing its vaporization [58].

### 2.2. Sputtering Systems

Sputtering is a physical process that involves bombarding a solid target material with energetic ions or atoms, typically using plasma (Figure 4), which causes the target material to eject atoms or molecules from its surface [59]. As a result of the reduced collision probability in a low-pressure environment, the ejected particles travel within the vacuum chamber with an almost straight-line trajectory [60], as confirmed by the good agreement between theoretical studies (mainly based on the Monte Carlo method) and experimental results [61,62]. The line-of-sight trajectory is responsible for the typical columnar microstructure of the deposit [63].

There are several variants of sputtering techniques, but the most common is called DC magnetron sputtering [35]. In this process, a negatively charged target material is bombarded with positively charged ions from a plasma in a low-pressure environment, typically made of an inert gas such as argon. The positive ions from the plasma collide with the target, causing atoms or molecules to be ejected. Recent developments in sputtering technology are summarized in the following paragraphs.

*High-Power Impulse Magnetron Sputtering* (HiPIMS) [64] is a magnetron sputtering process in which the plasma is generated through short and high-power current pulses. This technique has been shown to produce films with improved adhesion, higher density, and increased film quality compared with conventional DC or RF magnetron sputtering [65]. Keraudy in 2019 [66] investigated the effect on the ion energy of bipolar HiPIMS, where the standard HiPIMS pulse is followed by a reversed potential applied on the target, demonstrating that the amplitude of the reversed potential gives excellent control over the ion energies. This is particularly interesting in realizing insulating thin films, for which adjusting the energy of the impinging ions through the substrate’s bias voltage is not feasible. Tiron in 2019 [67] applied the bipolar regime to diamond-like carbon (DLC) films, increasing density and hardness. Brenning in 2021 [68] studied the electron discharge of insulating substrates by mixed high- and low-power pulsing. Ghailane in 2020 [69] reviewed the latest HiPIMS deposition system designs developed to improve the performances of hard coatings.

*Pulsed DC Sputtering* (PDMS) [70] is a DC sputtering technique that generates plasma with short, high-power current pulses. PDMS has been shown to improve film quality, reduce arcing, and improve target utilization. Dong in 2021 [71] studied the role of the frequency of the pulses (Fp) on the deposition of vanadium oxide thin films, finding that an increase in Fp resulted in a reduction of the deposition rate and a transition from the typical columnar structure to a smoother and fully dense deposit.

*Hybrid Sputtering* [72] involves combining multiple sputtering techniques, such as magnetron sputtering and ion beam sputtering, or bipolar sputtering with HiPIMS. For instance, high-density [73] and low-roughness [74] films can be obtained by combining magnetron and ion beam sputtering with the technique known as Ion-Beam-Assisted Deposition (IBAD) [73,74,75]. Concurrent different sputtering processes can lead to complex deposition mechanisms: plasma diagnostics and material-characterization methods may help clarify how plasma characteristics affect coatings properties. Among the operating parameters, frequency, duty cycle, substrate bias, total pressure, and reactive gas partial pressure are probably the most significant. The effects of the pressures and biases have been widely investigated [53,70,76,77]; however, there is still much to do about the frequencies and the duty cycles. Emile Haye in 2018 [78] investigated the effect of duty cycle and pulse frequency on the reactive bipolar sputtering efficiency, highlighting that high frequencies combined with short duty cycles are a good compromise to achieve ionization with a limited back attraction of the species. Another example of mixed application was performed by Ou in 2020 [79], depositing CrN/Si_3_N_4_ multilayer coatings using combined Deep Oscillation Magnetron Sputtering (DOMS) with pulsed DC magnetron sputtering, obtaining harder coatings with better cracking resistance. These recent developments in sputtering technology are improving the quality and performance of thin film coatings in a variety of industries. An interesting analysis of PVD-Magnetron Sputtering for industrial applications was made by Baptista and Silva [21]. They examined the latest technology improvements that allow one to obtain smooth surfaces with excellent mechanical, tribological, and adhesion properties operating at lower temperatures.

### 2.3. PVD + Galvanic Hybrid Systems

Its flexibility and adaptability make PVD suitable for fulfilling fashion market demands. Unlike conventional coating techniques such as electroplating, PVD can coat both metallic and dielectric substrates (e.g., plastics and ceramics) with a wide range of materials, including nitrides [56], carbides [80], and oxides [81]. In the fashion industry, high-end coatings are mainly applied to brass and steel to enhance aesthetics and functional properties. However, direct PVD coating, especially on brass, may not offer sufficient protection against corrosion, so a combination of galvanic and PVD coating is often needed [36].

Galvanic deposition [82] involves immersing the substrate in an electrolytic solution containing ions of the coating material, with the substrate acting as the cathode. When a current is applied, the coating material condenses on the substrate. Galvanic coatings offer numerous advantages, such as good adhesion, ductility, and a high level of control over coating thickness and morphology. When a power source is applied, reduction reactions occur at the substrate’s surface, which is covered. Galvanic coatings offer several advantages, such as good adhesion, ductility, and high control over coating thickness and morphology. Both PVD and galvanic coatings have advantages and limitations; PVD coatings are typically harder and more wear-resistant, making them ideal for high-wear applications. Compared with electroplating, even better wear resistance can be achieved using hard materials as underlayers [42]. They also offer a broader range of color options, including metallic and non-metallic ones. However, PVD coatings require complex equipment and are generally more expensive than galvanic coatings. Additionally, PVD coatings may be less ductile than galvanic coatings, leading to cracking or peeling under certain conditions.

**Figure 6:**
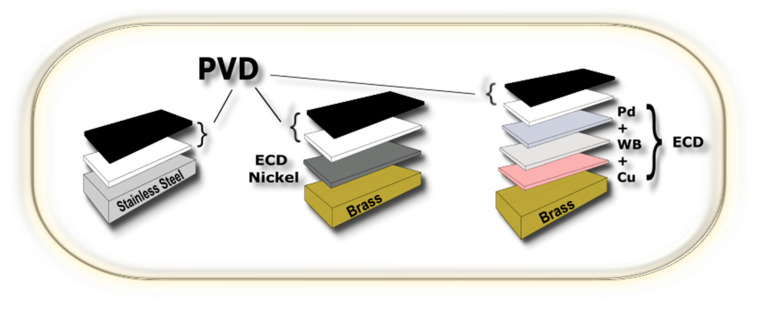
Schematic illustration of typical surface finishes for substrates such as stainless steel and brass.

On the other hand, galvanic coatings have lower prices than PVD coatings and can be applied to a wide range of substrates. They offer good adhesion and ductility, making them suitable for applications that require flexibility or deformation. Combining PVD and galvanic deposition can offer unique advantages for decorative coatings. It enables one to obtain a broad color range, improved adhesion, and enhanced wear resistance. The most straightforward hybrid process can be realized using an electroplated surface as the base layer for a PVD-made topcoat that determines the appearance of the finish. This approach provides numerous color and finish options and enhances the durability and wear resistance of the coating. An example of multilayer architectures for brass and stainless steel is shown in Figure 6. On the left side, stainless steel is covered by two PVD layers, the adhesion layer (in white) and the final PVD finish. In the center of the picture, a brass substrate is covered with an electrochemically deposited (ECD) nickel layer; a PVD topcoat is then realized using an intermediate thin film to improve its adhesion (adhesion layer in white). On the right side, a PVD topcoat is realized on a typical nickel-free ECD stack consisting of copper, white bronze (WB), and palladium. Another way to combine PVD and galvanic techniques is through so-called “graded coatings”, where the composition or texture gradually changes during growth, an approach that can improve the erosive, abrasive, and wear properties of the coating. An example of graded coatings realized by varying the current and the voltage bias during deposition was studied by Antonov [83].

The hybrid approach produces unique aesthetic effects and enhances the coating’s performance by tailoring its properties to specific substrate areas [36,84].

Overall, the combination of PVD and galvanic techniques provides a powerful toolset for creating decorative coatings with a broad range of colors, textures, and properties. By carefully selecting the deposition parameters and process conditions, it is possible to obtain coatings with enhanced adhesion, wear resistance, durability, and unique aesthetic features. On the other hand, the combination of two completely different deposition processes, such as electrodeposition and PVD, increases the production complexity and costs. For this reason, research is active to minimize the inconvenience of the two techniques in order to obtain a single standalone process.

## 3. Substrates

**Figure 7:**
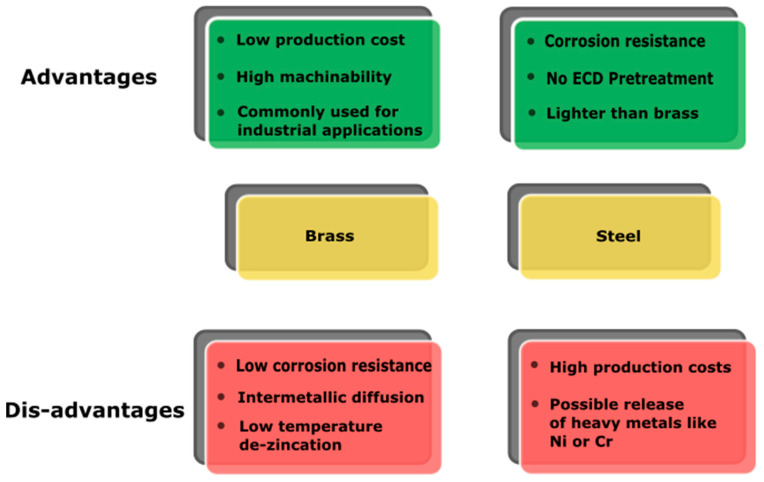
Schematic description of main advantages and disadvantages of brass and steel as substrates.

PVD coatings can be deposited on several substrates, such as, Ti, Al, Cu alloys, steel, and even plastics (e.g., polycarbonate [85]). Corrosion-resistant substrates are preferred because PVD coatings’ corrosion performances are highly dependent on the microstructure. The well-known columnar structure, typical of PVD films, is due to the line-of-sight process, often associated with a not negligible degree of porosity. Voids within the coating could be the starting points for substrate degradation processes [86]. This is just one of many aspects to keep in mind when choosing substrate material for industrial applications. The compatibility with PVD processes is an often-overlooked aspect in base material selection. The main drivers are the raw material cost, the ease of working, and the suitability for large-scale manufacturing. This section will discuss two of the most common substrates for decorative applications, i.e., brass and stainless steel. The benefits and drawbacks of using brass and steel as substrates in industrial deposition processes are summarized in Figure 7.

### 3.1. Brass

**Figure 8:**
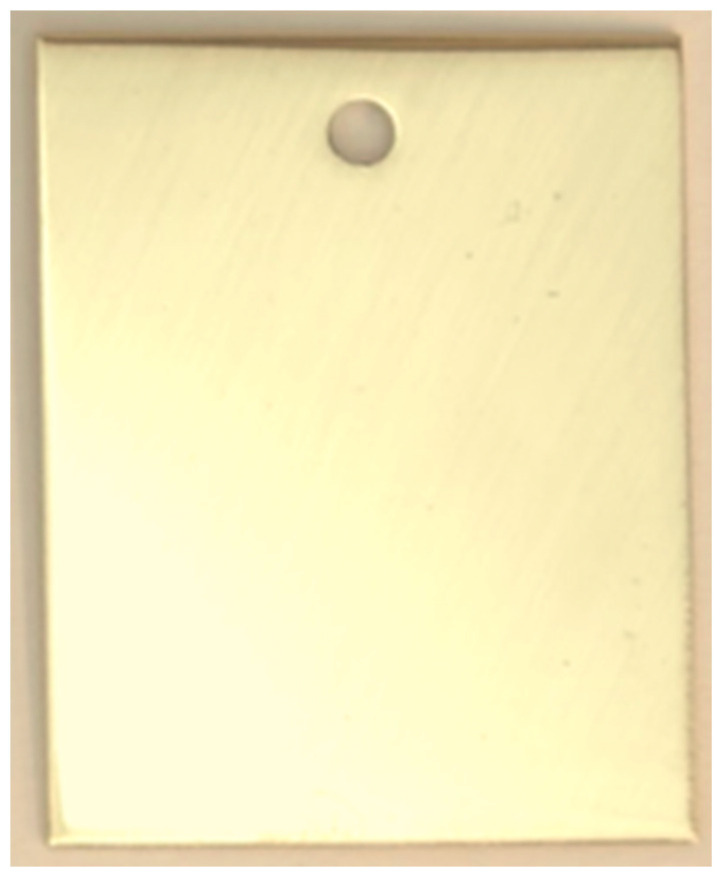
Brass coupon with polished finish.

The term “brass” (Figure 8) refers to various alloys containing copper and zinc as the main components. Thanks to its forging and machinability properties, brass is the most employed substrate for decorative applications. Fashion accessories are usually made through hot stamping [87], a low-cost and highly adaptable processing technique. Dual-phase (α + β) and leaded brasses are preferred because of their better machinability [88,89,90,91,92]. Lead-free brasses containing silicon and bismuth also have good machinability and can be used as decorative substrate materials [91]. From a practical point of view, Si-containing brasses, due to their poor electrical conductivity, are not recommended if electrochemical pre-treatments are required. PVD on brass is usually preceded by an ECD [32,93] of an anticorrosion and a leveling layer to increase lifetime and brilliance. ECD pre-treatments are needed because PVD does not allow for the sealing of the substrate; external agents can, therefore, come in contact with the substrate, promoting its corrosion, delamination, and intermetallic diffusion processes [94]. An ECD of bright nickel is commonly used under a PVD coating as it provides good anticorrosion and leveling properties. An ECD of copper is used as a leveling layer for nickel-free products, while white bronze and palladium layers work as anticorrosion and diffusion barriers [95] (Figure 6).

**Figure 9:**
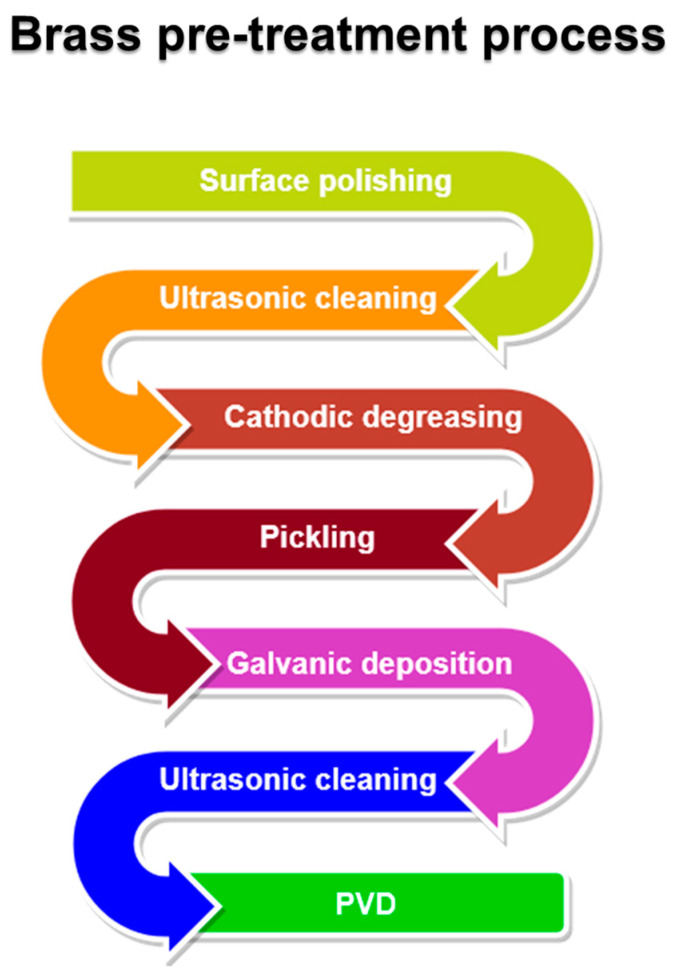
A flowchart representing the pre-treatments to which brass is subjected in a coating process consisting of both electroplating and PVD.

Before electroplating, brass substrates are polished, cleaned, degreased, and the superficial oxides are removed: this preparation is obtained with different steps involving ultrasonic cleaning in an alkaline solution at 60 °C, followed by electrochemical alkaline degreasing and cleaning in diluted sulfuric acid [82]. If Si-based brasses are used, rinsing in hydrofluoric acid between ultrasonic cleaning and degreasing is advisable to improve the adhesion of the deposit. Ultrasonic cleaning is usually performed after electrochemical treatments before entering the vacuum chamber (Figure 9). Some studies of direct PVD on brass for decorative purposes are present in the literature [84,96,97], but as far as the authors know, those procedures are not used in industrial applications.

### 3.2. Stainless Steel

**Figure 10:**
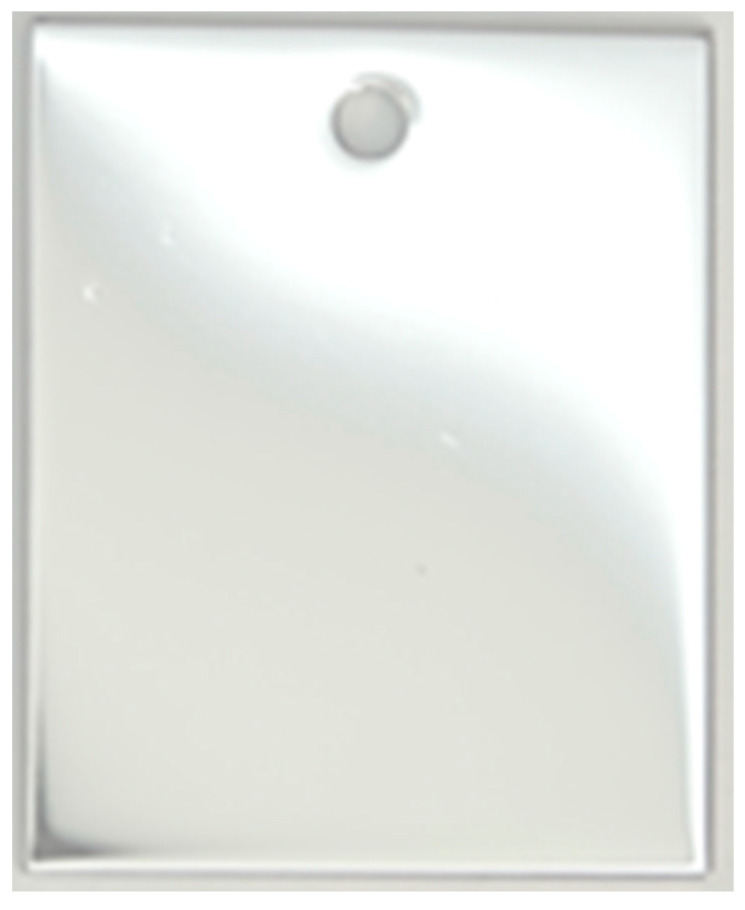
AISI 304 coupon with polished finish.

The generic name “steel” represents a vast collection of iron-based alloys containing carbon whose composition has been developed according to the needs of specific applications. European standard EN 10027 [98] classifies steel alloys by a nomenclature representing their physical and mechanical characteristics. One of the most common designation systems is the AISI (American Iron and Steel Institute), developed by the homonymous organization. The AISI identifies steels by a three-digit code followed by an optional letter. AISI 304 is the most popular steel for high-end fashion accessories (Figure 10).

The performances of steels depend on the properties associated with their microstructures, i.e., the composition, the size, the morphology, and the arrangement of the constituting phases [99,100,101,102]. Since all phases in steels are crystalline, steel microstructures are composed of various crystals, sometimes up to three or four different types, that are physically mixed during phase changes and heat treatments. Among all microstructures, the most relevant and studied are the ferritic, martensitic, and austenitic types. Ferritic steels are ferromagnetic and have a high temperature resistance. Martensitic and precipitation steels contain low amounts of nickel and molybdenum; also, these types of steels are magnetic and have high hardness and toughness. Austenitic steel is the largest group of steels and can be subdivided according to the chemical composition in (i) Cr-Mn; (ii) Cr-Ni; and (iii) Cr-Ni-Mo. Every alloying element actively empathizes a specific physical or chemical property, as reported in the *Handbook of Stainless Steel* by Outokumpu Oyj company [103].

In addition to iron, the main components of steel are carbon, chromium, and nickel.

*Carbon*—It promotes the austenitic microstructure, increasing mechanical strength. However, it has a negative impact on the intergranular corrosion resistance caused by carbonate formation [104]. This has led to the development of low-carbon alloys. In ferritic alloys, carbon greatly reduces the breaking stress and corrosion resistance. In martensitic alloys, it increases hardness and strength but decreases toughness.

*Chromium*—It is a transition metal, hard and brittle, with strong corrosion resistance. Chromium is highly prone to combine with oxygen, producing a passivation film of stable oxide that protects the surface from corrosion; therefore, it is always used in producing stainless alloys. It accounts for at least 10 wt% in stainless steel and the corrosion resistance increases almost linearly with its abundance. Chromium also increases resistance to high-temperature oxidation, promoting ferritic microstructure formation.

*Nickel*—It promotes the formation of the austenitic microstructure, as well as provides greater hardness and ductility. In addition, it reduces the corrosion rate in the active state and improves resistance in acidic environments. In precipitation-hardened steels, nickel forms intermetallic compounds which increased strength, while in martensitic structures, it is combined with carbon to improve weldability.

The performance of steels depends on the properties associated with their microstructures, that is, the arrangements, volumetric fractions, sizes, and morphologies of the various phases constituting a macroscopic section of steel with a given composition in a particular processing condition [99,100,101,102]. Unlike brass, many publications report direct PVD coating on stainless-steel substrates [105,106,107,108,109].

**Figure 11:**
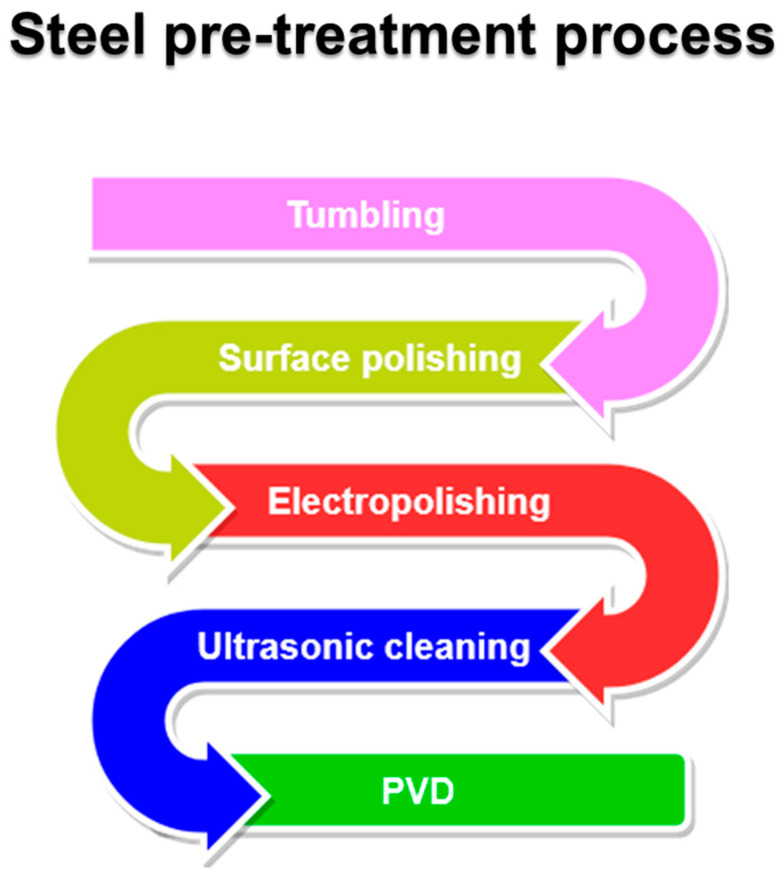
A flowchart about steel surface pre-treatments before entry inside the PVD chamber.

Superficial defects and imperfections created during the manufacturing process can interfere with protective film formation, reducing resistance to certain types of corrosion [110]. Concerning the PVD process, to obtain good coatings, the surface preparation process must be well-established and highly reproducible. The typical pre-treatment procedure consists of the following steps: tumbling, polishing, electropolishing, and ultrasonic cleaning (Figure 11). A more detailed discussion of stainless steel cleaning processes is reported in ASTM International Handbooks [111].

## 4. Surface Finishing

### 4.1. Titanium-Based Coatings

**Figure 12:**
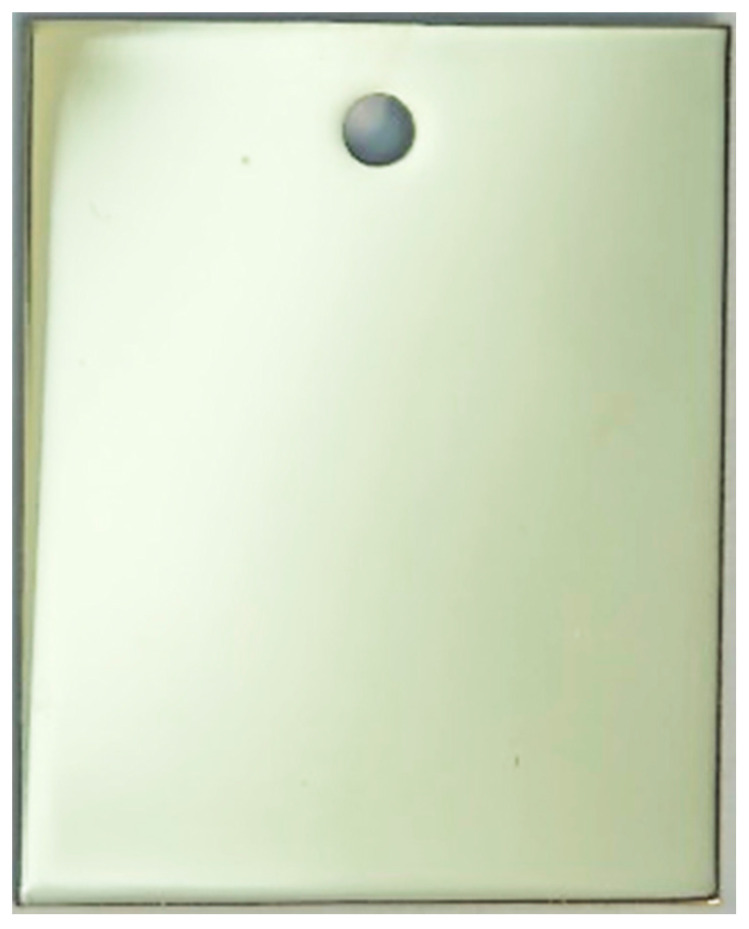
Example of a golden-colored TiN coating.

Decorative titanium-based coatings have been widely used in the industry due to their biocompatibility [112], color options [113], remarkable corrosion resistance [105], and cost-effectiveness. TiN coatings, as shown in Figure 12, can be prepared by adjusting the vacuum to 5 × 10^−5^ Torr, heating the substrate to 100 °C, and using 4 A of input current. In these conditions, nitrides like TiN [114] and ZrN [113] can be deposited; these have been extensively researched for a variety of industrial applications such as hard coatings [105], diffusion barriers in semiconductor technology, mirrors for optical applications [115,116], and decorative coatings.

Several factors influence the deposition of TiN coatings, such as the reactive gas used, the total pressure in the chamber [117], Ar/N_2_ ratio [118], substrate temperature [119], and the substrate’s bias voltage [120,121]. Among these factors, nitrogen flow is the most crucial because it determines the color of the coatings. The deposition conditions that produce the brightest yellow must be chosen precisely. For instance, a mistaken N_2_ supply can produce unaesthetic dull colors [122]. The watchmaker industry developed the historical process for gold coating, which involved covering a thin layer of titanium nitride with a flash of gold to create a highly wear-resistant coating. Combining TiN and ZrN layers, a gold-like appearance has been successfully obtained in producing luxury pens [123].

Carbides are prepared similarly to nitrides, using acetylene instead of nitrogen as a reactive gas; they are an alternative to the most expensive DLC for the realization of black coatings, albeit with lower hardness and stress resistance. To overcome these drawbacks, Gupta in 2019 [124] proposed a new type of black coating, i.e., TiAlCO, using ion implantation. The Ti surface was bombarded with energetic carbon ions, applying 2 kV anode voltage and a 0.03 T magnetic field. A carbon-rich plasma was established at 2 × 10^−6^ h·Pa and a minimum ion fluence of 10^18^ C·cm^−2^ was necessary to achieve a black color surface.

Blue-colored coatings based on TiNO or ZrNO can be obtained by increasing the oxygen content in the nitride lattice. Another well-known material for decorative applications is TiO_2_, thanks to the wide range of colors obtainable by varying the coating thickness [81].

### 4.2. Chromium-Based Coatings

Chromium is a popular choice for PVD coatings due to its excellent mechanical, thermal, and chemical properties [125]. Chromium coatings are widely used in a variety of industries, including the aerospace [126], automotive [2], and medical industries, due to their ability to provide high wear resistance, hardness [110], and corrosion resistance [127].

**Figure 13:**
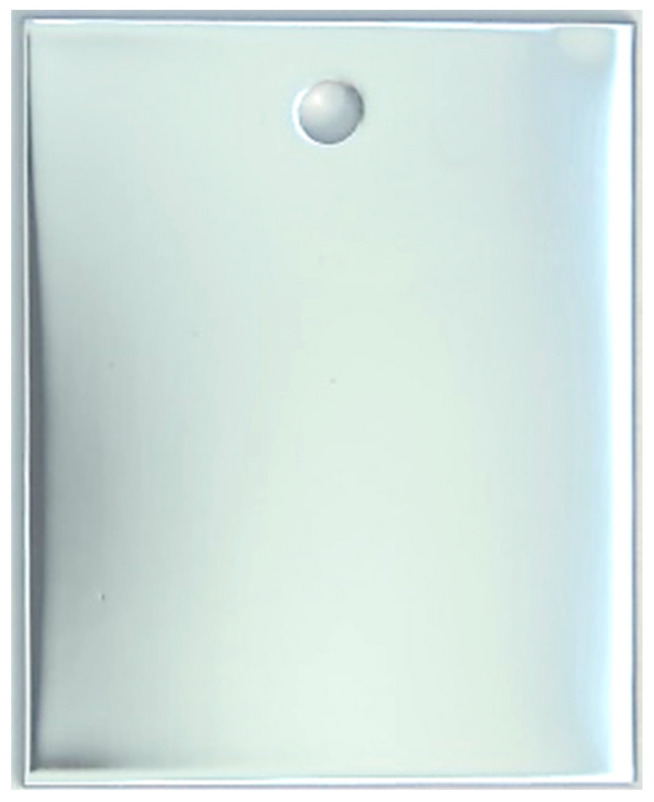
Example of a gray Cr coating.

Chromium PVD coatings (Figure 13) are known for their exceptional resistance to wear and abrasion, making them ideal for use in applications that involve high levels of friction or impact. Additionally, chromium coatings have a low coefficient of friction, which makes them ideal for use in applications where sliding or rolling contacts are involved [128], such as bracelets and anklets.

Another advantage of chromium PVD coatings is their ability to provide excellent corrosion resistance. Chromium is easily passivated, which means that chromium coatings can protect the underlying material from rust and other forms of corrosion. It is also worth noting that chromium PVD coatings can come in a range of different colors, from a bright silver color to a darker gunmetal gray. The color of the coating can be controlled by adjusting the deposition conditions, such as the gas mixture, pressure, and temperature. Chromium PVD coatings are of interest to the decorative industry as an alternative to ECD chromium. Shiny and highly reflective ECD chromium coatings are widely used and appreciated; those are generally obtained via electrodeposition from Cr(VI) baths. Hexavalent chromium is a known human carcinogen, and its use has been heavily restricted by the European Union [129]. PVD chromium competes with Cr(III) baths to fill the space left in the market by Cr(VI) baths, as trivalent chromium baths have considerable criticalities such as the high temperature and high voltage required and worse mechanical and aesthetic properties due to the presence of carbides formed during the electrodeposition. In 2022, Martinuzzi [36] published a comparative study between PVD chromium and ECD chromium, proving how PVD chromium coatings are a valid alternative to ECD deposits obtained from Cr(VI) solutions. He deposited Cr PVD on a copper substrate at 6.3 × 10^−3^ Pa pressure, heating the substrate at 300 °C, while the plasma current was set at 150 mA.

### 4.3. Zirconium-Based Coatings

Zirconium-based ceramic compounds are used as a top layer due to their peculiar mechanical and aesthetic capabilities; an exhaustive review of Zr(N, C, CN) properties was made in 2020 by Ul-Hamid [130]. The most famous Zr-based ceramic for decorative applications is ZrN thanks to its golden-like color. Already in the late 1980s, ZrN-based coatings were first investigated as a hard coating for industrial applications such as wear-resistant protective layers [131]. ZrN PVD coatings exhibit a wide range of color depending on the deposition parameters, ranging from yellow to silver and brown to gray. In the late 1990s, it was clear that, unlike TiN, the color was dominated by the microstructure and not by the stoichiometric composition [93] and it could achieve a higher brightness (L* > 90) [132]. Nitrogen partial pressure is fundamental for color control, and the gold-like color was obtained with a N_2_ flux of 8–10 sccm. In 2001, Nose [113] proposed a comprehensive explanation of how the color is influenced by deposition parameters. Then, in 2020, Ul-Hamid [133] made a complete review of deposition conditions that influence the mechanical properties of various Zr ceramic compounds. From a decorative perspective, it is interesting to note that the golden color of ZrN is more greenish than the one obtained with TiN, but the higher brightness makes ZrN more suited for Au + ZrN systems. It has been proved that with the wear of the top-deposited gold layer, a close matching in L* guarantees a more pleasant aesthetic effect than a satisfying matching in hue [20]. Klumdoung [134] obtained silver, brown, green-yellow, and blue ZrN deposits working at a high Ar flow rate (6 sccm) and varying the nitrogen flow from 0 sccm to 6 sccm, again reporting the correlation between color and crystal structure. The corrosion resistance properties of ZrN deposits are strictly correlated to structural parameters such the grain size [132], and according to Kuznetsova [135], deposit structure can be accurately tuned by optimizing the N_2_ flow. For decorative applications, multilayer Zn/ZrN systems are preferable due to their superior corrosion-resistance properties [136,137]. Multilayer ZrN/TiN systems can produce coatings with high hardness and stronger adhesion [138] compared with ZrN and TiN systems. Recent works have focused on improving ZrN’s mechanical and optical properties with the addition of Si and O as ternary elements [139,140,141]. Furthermore, to obtain coatings with antimicrobial capabilities [142,143], the addition of Cu to Zr-based ceramics has been evaluated. Gray PVD coatings can be achieved by depositing ZrC [144], but this top layer is uncommon in the decorative field since Cr-based coatings are preferred.

### 4.4. DLC Coatings

**Figure 14:**
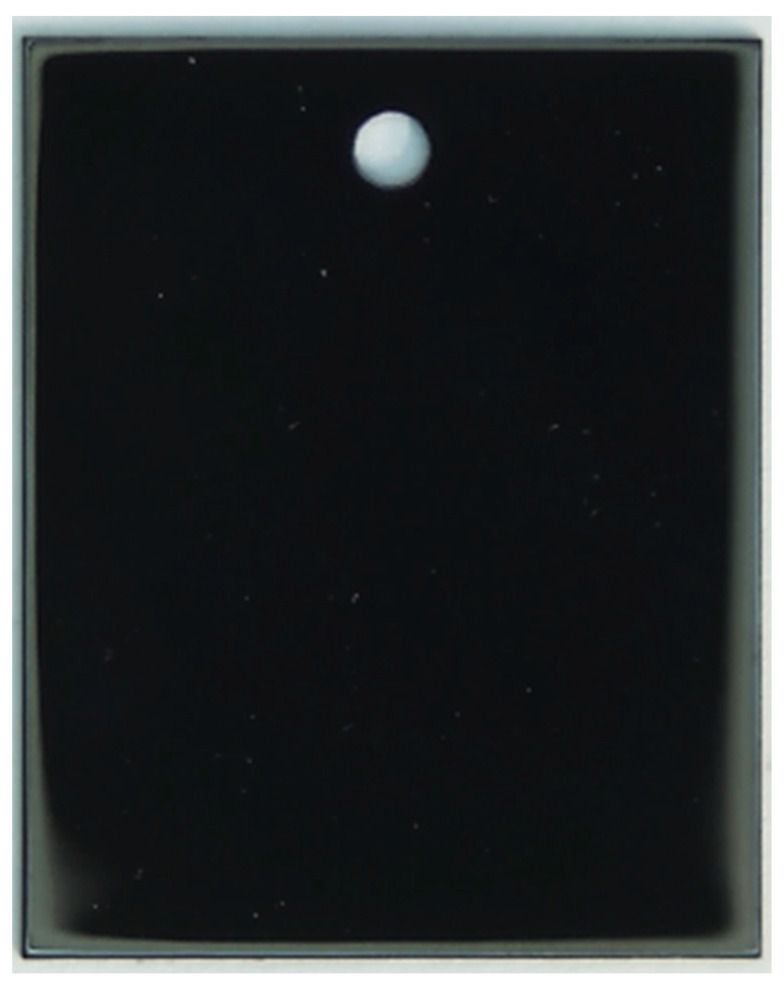
Example of a black-colored DLC coating.

DLC coatings are amorphous carbon-based materials containing a mix of sp^2^ and sp^3^ hybridized C atoms. Their unique properties, such as high hardness, low friction, high thermal conductivity, and chemical inertness, rank among the two allotropic forms of carbon (graphite and diamond) and they depend on the sp^2^:sp^3^ ratio [145]. Raman spectroscopy is fundamental to characterize DLC: from D and G bands; it is possible to estimate the sp^2^:sp^3^ ratio and the internal stress [146,147]. DLC can be deposited by PVD using a graphite source and by CVD using a mixture of hydrocarbons as reactive gas [148]. The electrodeposition of DLC on Ti was achieved in 2009 by Manhabosco [149], but required a rapid increase in applied potential from 0 to 1200 V for 4 h and acetonitrile mixed with DMF as a medium; for their simplicity, PVD and CVD remain the best way for industrial applications. An in-depth analysis of DLC properties and classification was made in 2021 by Ohtake [150]. Due to their tribological and chemical properties, DLC coatings are widely used for cutting tools, engine parts, optics, and corrosion barriers, as reported by Vetter [151] in their historical review of DLC deposited by PVD for industrial applications from the first developments to 2014. DLC deposits are interesting for decorative applications because their black color (Figure 14), low friction, and wear resistance make DLCs great to be used as a finish on bracelets and watch bands [152]. DLCs are promising as a corrosion barrier too, but due to their high internal stress they are not suited to be an intermediate layer [144]. Low adhesion and delamination are the main problems that limit the usage of DLC in decorative applications. To improve the adhesion on a steel substrate, a Cr-based underlayer system was developed by Duminica in 2018 [43], and in 2021 Gómez [153] obtained a DLC coating on steel with surprising adhesion deposited with high-power impulse magnetron sputtering (HIPIMS) technology with positive pulses. For the DLC film, they used a graphite target operating at 600 V and 1.5 A.

## 5. Characterization of PVD Coatings

In decorative and high-end fashion industries, coating defects could compromise aesthetic properties, which are crucial in determining the commercial success of a product [154]. For this reason, accurate quality control planning of quality control is mandatory to reduce rejected products and increase company competitiveness [155]. A standard quality control protocol for PVD coatings has to include the evaluation of tribological properties (at least hardness and wear resistance), adhesion tests, corrosion resistance, and color measurements.

This section provides a comprehensive overview of the most common tests and analyses implemented by quality control processes adopted in decorative finishing.

### 5.1. Thickness Determination

Probably the most common analysis in the decorative industry for PVD coatings is thickness determination [156]. Thickness is an important parameter, and it is correlated with other properties like adhesion, wear and corrosion resistance, and barrier properties [157,158]. The importance of layer thickness is amplified in modern multilayer systems, as their properties depend on the interface volume between layers [159,160]. As other important parameters for quality control, the thickness measurement methods of multilayer systems go under ISO 21874:2019 [161]. Thickness determination methods can be subdivided into destructive and non-destructive techniques. To examine multilayered structure methods involving Scanning Electron Microscopy (SEM) analysis, metallographic cross sections are preferable because they allow one to directly visualize and measure the multilayered structures. Other popular destructive techniques are the crater grinding method (regulated under ISO 26423:2016 [162,163]) and GDOES [164]. Thickness measurements methods based on X-ray fluorescence (XRF) and regulated under ISO 3497:2000 [163] are the most popular in industrial applications: they provide fast and accurate analysis, especially for metallic coatings, and various desktop ED-XRF, coupled with user-friendly software, are sold on the market for industrial applications. A new trend is the automatization of XRF measurements and the capability to obtain real-time data during the deposition [165] to improve further the quality control over the products. A standardless approach based on Monte Carlo simulations for XRF was proposed in 2019 [166]. Indirect thickness measurements with quartz microbalances (QCM) are also popular to perform real-time quality control on the deposited material over the surface [158]. To evaluate the thickness of thin films, Giurlani [167] developed in 2018 a standardless method based on EDS spectroscopy and Monte Carlo (MC) simulations: it was able to measure nanometric thicknesses of PVD-sputtered samples. In 2020, MC simulations were also employed with success to make calibration curves for XRF thickness measurements [168]. Another emerging non-destructive methodology was proposed in 2021 by Isern [169]; it is based on terahertz (THz) reflectivity and was successfully employed to map the PVD-deposited yttria-stabilized zirconia thermal barrier. In 2023, Cruz [170] proposed a standardless method, tested on TiN coatings, based on EDS and MC simulations that correlates the acceleration voltage, the type of substrate, and the intensity ratio of peaks of the substrate and the deposit to the coating thickness.

### 5.2. Mechanical Properties and Defects Analysis

Mechanical and tribological properties have been the subject of numerous studies [56,151,171,172,173,174,175]. In decorative PVD coatings, tribological properties play a critical role in determining the resistance of the coating to wear and abrasion, because they can affect the appearance and the lifespan of the coating. Therefore, coatings with good tribological properties such as high hardness, low coefficient of friction, and good wear resistance are preferred for decorative PVD applications. Several tribological parameters such as coefficient of friction, wear rate, and volume are evaluated with a pin-on-disk Tribometer; this test is regulated by ASTM G99-17 [176], DIN 50324-07 [177], and ISO 18535:2016 [178]. Hardness and adhesion are other important mechanical properties to evaluate the quality of a produced good: accessories and fashion jewels are subjected to continuous changes in temperature and frequent shocks and bumps. Small detachments or scratches that compromise the aesthetic value of the object determine the end of life of the product, as decorative goods lose functionality as soon as the visual appearance worsens and it is no longer desirable. Then, to improve the overall quality and reduce the embodied energy (the total energy required to produce a product, from raw material to the delivery of the final good), decorative PVD coatings need to pass hardness and adhesion tests. For hardness evaluation, indentation [179] tests are regulated by ISO 14577-1:2015 [180]. The Rockwell adhesion test is the one of choice to evaluate the adhesion of PVD coatings, as it provides quantitative information and it is regulated under ASTM C1624-22 [181] and VDI 3198 [182]; developments on automatizing and standardizing the Rockwell test involving neural networks and machine learning algorithms are ongoing [183]. Other adhesion tests are the network of cuts method under ISO 2819:2017 [184] and ISO 11644:2022 [185] and the tape test under ISO 11644:2022 [185]. A novel methodology, based on the crater grinding method and adhesion scratching tests, to obtain quantitative information on deformations and degradation of PVD multilayer films named the Recatest was proposed in 2021 by Domanowski [186]. Defect analysis is fundamental for PVD coatings due to the columnar structure and poor coverage of the deposits, and an excellent review of PVD growth defects was written by Panjan [86] in 2020. To differentiate defective products that could accelerate degradation processes and ensure the customer receives a high-quality product, the techniques to analyze PVD deposits, especially multilayer ones, are regulated under ISO 21874:2019 [161]; the election techniques are SEM analysis and Glow-Discharge Optical Emission Spectroscopy (GDOS) [187,188].

### 5.3. Color Evaluation

Color is probably the most important parameter for the haute couture and decorative industries: the main aim of a finishing coat in those fields is to provide aesthetic value to the artifact, increasing the perceived value, to make it a luxury and desired object. Several studies have been conducted on the importance of color and its implication in marketing and psychology [189,190], and the link between a good quality PVD coating and the precise color requested by a customer is so tight that it is mandatory to have a standardized and quantitative method to define, measure, and classify colors. Environmental factors (e.g., the source of illumination) and the intrinsic properties of objects can generate inconsistent data, causing disputes between manufacturers and customers. To avoid this, the color is usually encoded through the standard dictated by the L*a*b* color space (also known as CIELAB or CIE1976). The CIE (Commission International de l’Eclairage) establishes the procedures, lighting sources, and observation angles that can be used; those procedures are under ISO/CIE 11664:2019 [191]. The values of the three coordinates L*a*b* are obtained through the transforms relating to the X, Y, and Z coordinates of the color space CIE XYZ [192] and the mathematical treatment is already covered extensively in the literature [193]. The L*a*b* color space covers the entire gamut of the visible human spectrum and can be understood and represented as an opposite color model with b* that shifts from yellow (b* < 0) to blue (b* > 0), a* from green (a* < 0) to red (a* > 0). L* represents the brightness and goes from 0 (pure black) to 100 (pure white). Colorimetric measurements can be achieved with a cost-efficient tristimulus colorimeter [194], but modern colorimetric spectrophotometers are better suited to obtain accurate data and avoid disputes with the customer [195]. The accuracy required for the color of a PVD coating is usually determined by the customer in the form of a* ± Δa*, b* ± Δb*, and L* ± ΔL, but an important parameter to evaluate the quality of a product is the Euclidean distance (ΔE) inside the L*a*b* color space (1)

(1) $$∆E=\sqrt[{2}]{{\left({∆L}^{*}\right)}^{2}+{\left({∆a}^{*}\right)}^{2}+{\left({∆b}^{*}\right)}^{2}}$$

A just noticeable difference (JND) between two objects is detected by a human eye for ΔE ≥ 3 [196]. That threshold should be considered in the decorative PVD industry to evaluate the production quality.

### 5.4. Corrosion Tests

As mentioned in previous sections, the aesthetic properties of a PVD coating in the decorative industry are the main task to obtain and maintain. Visible corrosion-derived defects, even if they do not alter the mechanical properties of the goods, determine the end of life in the decorative field [20]. Produced goods, to meet customer acceptance, have to pass various tests that simulate and accelerate the environments where those objects are going to be used [197] (e.g., the atmospheric conditions of a bathroom, especially for faucet industries); especially for wearable products, they need to pass tests that simulate human beings (e.g., sweat). Corrosion tests and corrosion resistance properties are extensively indagated for PVD coatings [36,56,171,198,199]. In the decorative industry, a commonly used substrate is brass, and corrosion studies are needed to evaluate the performances that could be compromised by the columnar structure of PVD [84,97,188]. The most used regulated tests for quality control in the decorative industry are the salt spray test, divided into neutral, acetic acid, and copper variants (ISO 9227:2022 [200]); the synthetic sweat test (ISO 3160-2:2015 [201] and NF S 80-772:2010 [202]); electrochemical impedance spectroscopy tests (ISO/TR 16208:2014 [203] and ISO 16773 [204]); potentiostatic and potentiodynamic polarization measurements (ISO 17475:2005 [205]); tests that simulate pollution and corrosive atmospheres (thioacetamide test ISO 4538:1978 [206], sulfur dioxide and nitric acid tests regulated under ISO 4524:2000 [207]); and dump heat (with leather, ISO 17228:2015 [208], and without leather, ISO 4611:2010 [209]). For haute couture industries, damp heat with leather is important to simulate the contact with chrome-tanned leather: it has been proved that chrome-tanned leather releases oxidating agents in the form of Cr(III) and Cr(VI) [210,211] that could damage PVD-covered accessories.

### 5.5. Heavy Metals’ Release

Regarding wearable artifacts, the release of heavy metals is a fundamental parameter for ensuring the quality and safety of the product for the customer. Common hazardous metals in wearable PVD-coated artifacts are nickel and lead. Although lead and nickel thin films can be deposited by PVD [212,213], those coatings are not of interest for decorative applications, but both could be found in small quantities inside substrate alloys. Nickel-containing steel is the most used type of steel for PVD applications and lead is added to brass to increase its machinability [214], even if the trend is to employ new performing lead-free brasses [91]. In addition, as we mentioned before, electroplated nickel is widely used as a strike layer for PVD topcoats if brass is used as a substrate. Nickel is considered a hazardous element for human health and its issues concerning human health are well reported in the literature [215,216]; the most common problem related to nickel-containing wearable objects is nickel allergy [217]. Nickel-release tests have to be carried out according to EN 12472:2020 [218], which regulates methods that simulate the wear and corrosion of artifacts for the detection of nickel release; nickel determination is regulated under EN 1811:2015 [219] and an accurate report of the techniques used to quantify nickel release in decorative industries was accomplished by Giurlani [95]. Lead needs to be considered one of the most important toxic heavy metals in the environment, and its full spectrum toxicity is reported in a 2015 review by Wani [220]; in the European Union, lead percentage in alloys is regulated by the European Chemical Agency (ECHA) and only alloys with lead content of a weight lower than 0.05% are admitted in jewelry. Other important regulations are the US standard ASTM F2999-19 [221] for adult’s jewelry, which sets a 1.5% threshold for lead in alloys, and the US standard ASTM F2923-14 [222] for children’s jewelry, which sets the threshold at 0.01% [223]. Determination of lead content in a sample is regulated under CPSC-CH-E1001-08.3 [224], ISO 26482:2010 [225], and EPA 6010C:2014 [226].

## 6. Recent Trends and Perspectives in the Decorative PVD Industry

The decorative industry, as highlighted by Bandinelli [4] in 2021, is characterized by low predictability and high variance in the types of artifacts produced and, particularly, in color requests. PVD in the decorative field has not yet succeeded in undermining the dominance of electroplating. Nonetheless, it is considered a fundamental technology for this sector, especially since it enables a wide range of colors and textures [227]. Although it is still a technology that is, at best, complementary to electroplating, it is gaining a lot of interest from the world of large-scale distribution mainly because of the possibility of obtaining more sustainable products, especially from an ecological point of view, meeting the requirements of the UN 2030 agenda [228]. The advantages of PVD compared with electroplating are well-reported in the literature [4,84,136,229,230], and the process improvements of recent years are well-reviewed in an excellent review from 2018 by Baptista [21]. The challenges that will have to be faced and solved to supplant ECD techniques are several: reduce structural defects as columnar growth, improve the research of PVD-deposited barrier layers toward intermetallic diffusion, and bypass the line-of-sight deposition limit. Regarding PVD barrier layers, research in the decorative field should take its cue from the realm of electronics. This is because the study of barrier layers to prevent copper diffusion is fundamental to ensuring the correct functionality of Cu interconnects [231]. A review made in 2020 by Li [232] underlined how the Ta/TaN barrier layer is state-of-the-art for Cu interconnects and how PVD-deposited Ru could be a valid candidate as a barrier layer, but the typical defects of PVD films such as the columnar structure limit its usage. Amorphous carbon obtained via DC magnetron sputtering as a promising barrier layer for Cu diffusion was proposed by An [233] in 2020. A complication in the design of barrier layers is the evaluation methodology, as a standard method of evaluation is lacking: to overcome this limit, in 2023 a novel technique based on X-ray microanalysis suited for the decorative and fashion industries was proposed [234]. The line-of-sight deposition limit is intrinsic to PVD, and with an engineering-like approach (substrate rotation, multi-target systems, and others) it is necessary to bypass that [173]. Many studies in recent years have been conducted to evaluate how the deposited microstructure changes in the function of the substrate angle and substrate oscillations [235,236,237]. Then, to meet the demand of the market, researchers should focus on making a wider range of colored deposits to cover the full color range offered by ECD; most of the obtainable PVD colors were well-summarized by Alliot [238] in 2023. To better understand and optimize PVD industries, in silico studies are trending as they can further reduce the environmental impact of the whole process by predicting properties [239]. Computational strategies can be divided into one that adopts an engineering-like approach and a computational chemistry one. Engineering-like approaches based on the Finite Element Method (FEM) and Computational Fluid Dynamics (CFD) for PVD were well-described in a 2018 review by Pinto [240]. In 2019, Kubečka [241] presented a 2D fluid model of an ion PVD-based process that successfully predicts the coating uniformity and the antenna effect on a workpiece of peculiar geometry. In 2020, Wang [242] simulated the magnetically induced ion motion during a PVD deposition inside a tubular substrate; important considerations have been drawn, as the deposition efficiency inside the tubular substrate is influenced by the magnetic field. Controlling the ion motion by adjusting the magnetic flux makes it possible to deposit at different positions inside the substrate. Computational chemistry approaches have been successfully employed to describe PVD processes, and both ab initio and classical molecular dynamics have been used. In 2016, Xu [243] investigated the deposition mechanism at the atomistic level of a TiN PVD deposit, and in 2018 Li [244] unveiled experimental differences between CVD and PVD MoS_2_ deposition and generated guidelines for future defects engineering. In 2020, Guo [245] explored the oxidation properties of a Ti-Al-N deposit alloyed with quaternary transition metals. In 2021, Kang [246] studied the oxidation mechanism of a Ti_2_AlC protective coating with an ab initio approach, and in 2022 Gholizadeh [247] described the cracking mechanism of a multilayer Ti/TiN system.

## 7. Conclusions

Physical Vapor Deposition (PVD) has been discussed as a technique to produce coatings for the decorative industry. It has been applied in the fashion industry since the 1970s and is regarded as the natural substitute for electroplating. However, it still has a marginal role within the decorative industry, mainly due to its lack of compatibility with brass, one of the most common substrates in the fashion field. Another issue highlighted during the review is related to the line-of-sight deposition mechanism, which could make it unsuitable for covering objects with complex shapes. The line-of-sight growth mechanism is also responsible for the deposit’s columnar microstructure, which reduces corrosion resistance and promotes intermetallic diffusion. Nevertheless, the ever-growing interest in environmental sustainability could convince luxury brands to switch to PVD technology. Notwithstanding recent advances, electroplating is still one of the most environmentally impacting manufacturing processes [26,248]. As *Fortune Business Insights* report, the rapid increase in market shares confirms the growing interest in PVD coatings in the fashion and decorative industries. Experts predict the PVD market value will double in the next five years. With its wide range of colors and textures, PVD can also be combined with electroplating, allowing fashion designers to fully express their creativity.

This review has focused on the latest developments in PVD technology and their applications to the fashion industry. Recent studies focused on new hybrid PVD technologies have been presented. Applications of PVD coatings to substrates such as stainless steel and brass have also been examined, highlighting how PVD is generally performed as a standalone process on stainless steel, while it needs to be coupled with ECD for brass. The importance of computational simulations is underlined, as they provide a powerful tool to predict coating properties, reducing production waste. It has shown that Ti-based coatings are the most used due to their wide color range (from yellow to blue). ZrN is reported as the best alternative to gold in realizing coatings for high-end vogue applications, while TiN is better suited for brass-like finishing, which is appreciated in the faucet industry. This review also examined quality control processes used by companies operating in the decorative sector.

## Data Availability

Not applicable.
